# Supplementary material for: Impaired Retromer Function in Niemann-Pick Type C Disease Is Dependent on Intracellular Cholesterol Accumulation
Source: Int J Mol Sci. 2021 Dec 9;22(24):13256. doi: 10.3390/ijms222413256 (PMC8705785; doi:10.3390/ijms222413256)
Supplement: Supplementary file 1 [file ijms-22-13256-s001.zip › ijms-1472106-supplementary.pdf]

Supplementary figures:

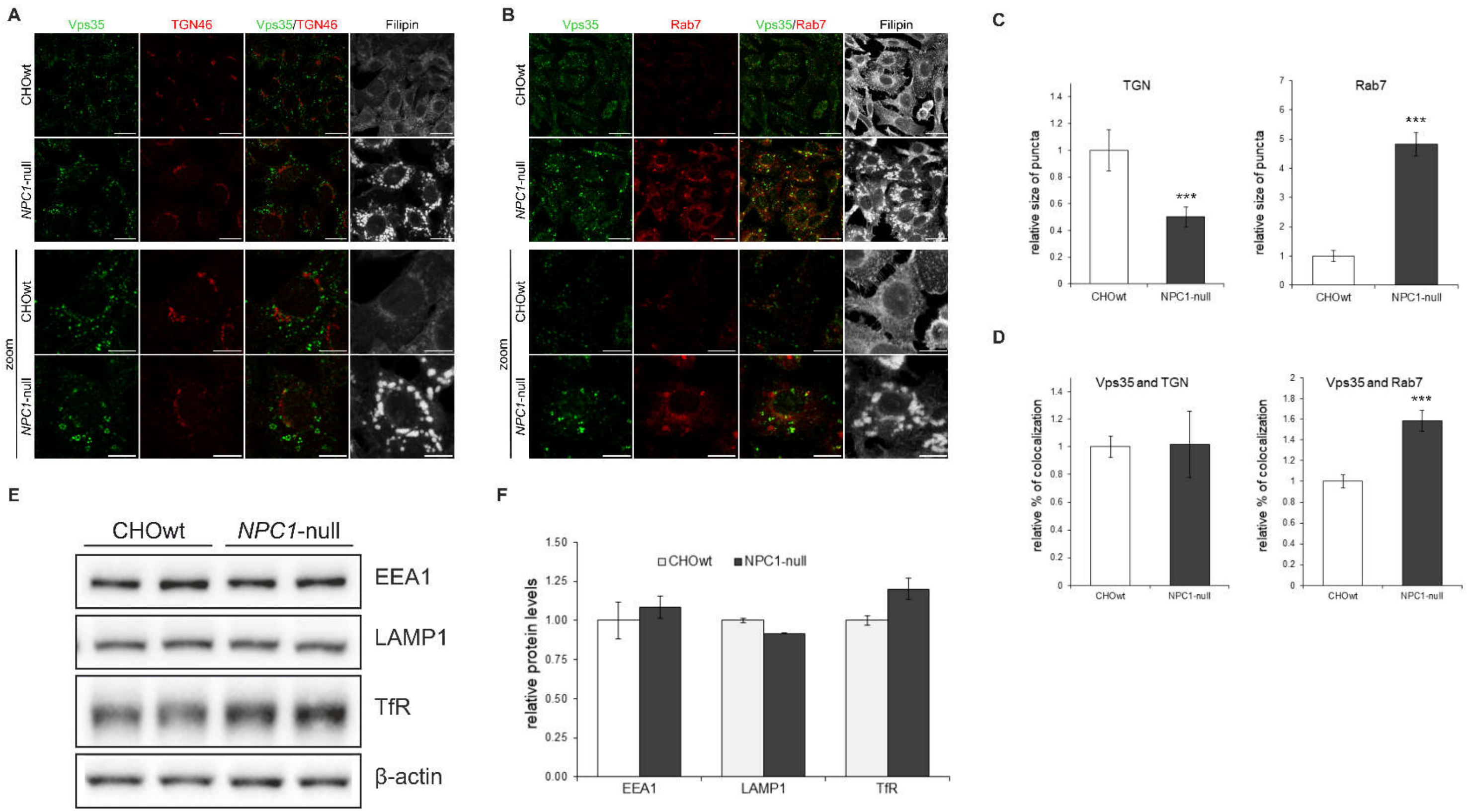

Figure S1. Vps35-coimmunostaining with trans Golgi Network and late endosome markers. The cells were grown in 10% FBS in DMEM/F12 media, fixed and stained for Vps35 (green) and A) *trans* Golgi Network marker TGN (red) and B) late endosome marker Rab7 (red). Filipin was used to stain free cholesterol. Cells were analyzed by confocal microscopy and signals were quantified using ImageJ software. C) Size of TGN and Rab7 puncta and D) colocalization of Vps35 and TGN or Rab7 between CHOwt and NPC1-null was compared, ***$p<0.001$. E) The protein levels of endolysosomal vesicles markers, EEA1, TfR and LAMP1, analysed by western blot, did not reveal any differences in the protein levels. Protein signals were quantified using the ImageJ software. The statistical significance of the tests was set at

p<0.05. The data are shown as mean ± SD normalized to control, n = 30 cells per group from three different experiments. Scale bar – 20 µm (unzoomed figures) and 10 µm (zoomed figures).

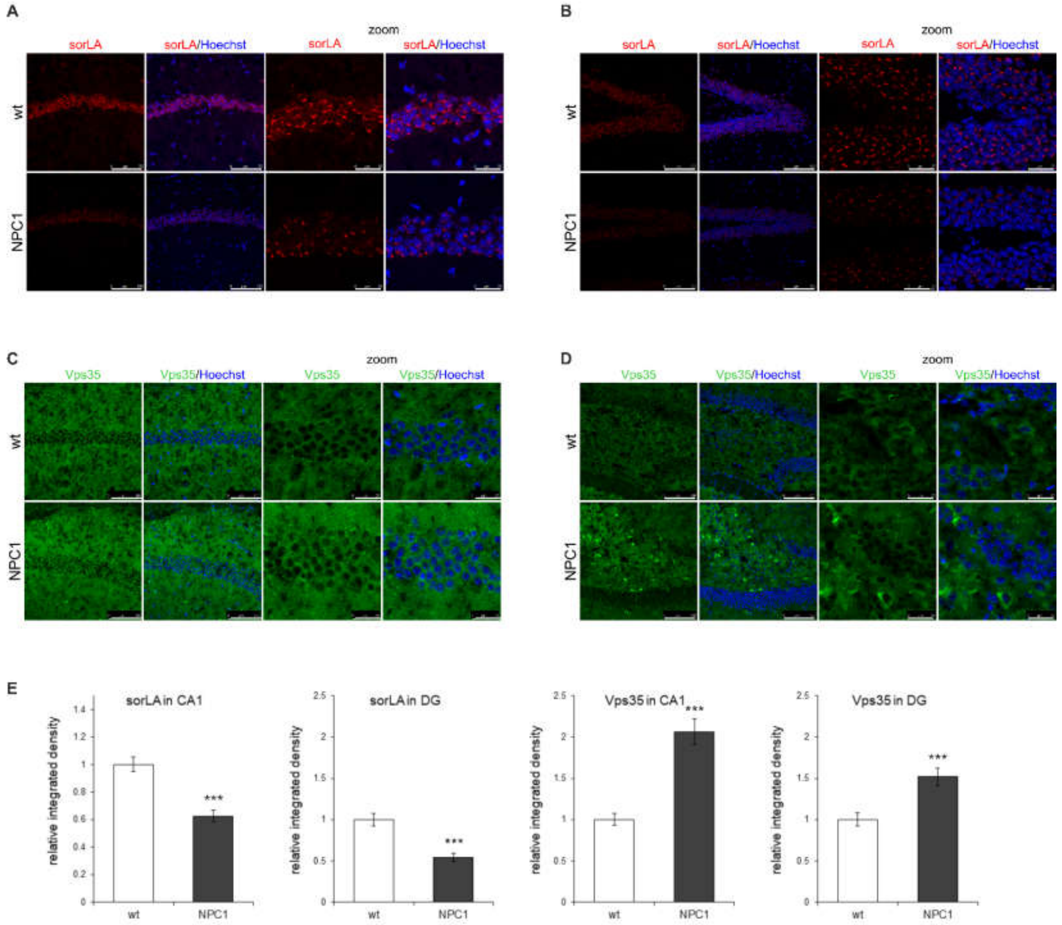

Figure S2. Immunohistochemistry analysis of retromer receptor sorLA and retromer protein Vps35 in CA1 and dentate gyrus regions of hippocampus in 10-weeks old wt and NPC1 mice. SorLA levels were analysed in A**)** CA1 and B**)** dentate gyrus (DG) and Vps35 levels in C**)** CA1 region and D**)** DG. While sorLa was distributed in the soma of neuronal cells of CA1 and dentate gyrus, Vps35 was localized in the neuronal soma of dentate gyrus' hylus. Immunostaining (integrated density) was quantified using ImageJ software. E**)** Decreased sorLA levels were detected in CA1 and DG neurons in NPC1-mice in comparison to wt, while, in contrast, Vps35 was accumulating in CA1 and DG neurons of NPC1 brains, ***p<0.001. The data are shown as mean ± SD normalized to control and represent data from three independent experiments, n = 9 slices per animal, six animal per group. Scale bar – 100 µm (unzoomed pictures) and 25 µm (zoomed pictures).

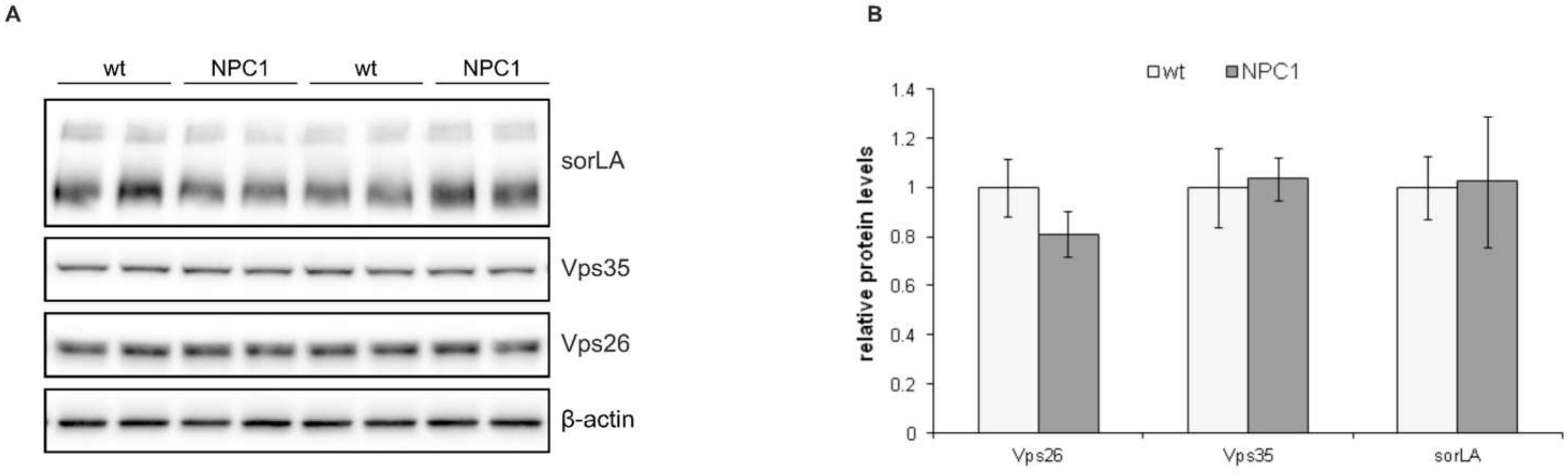

Figure S3. Altered retromer function in NPC1 mouse neurons is not due to changes in retromer proteins levels. The protein levels of retromer proteins Vps26, Vps35 and sorLA, analysed by western blot, did not reveal any changes between wt and NPC1 cortical neurons. β-actin was used as a loading control. Protein signals were quantified using the ImageJ software. The data are shown as mean ± SD normalized to control, from three independent experiments in technical duplicates. The statistical significance of the tests was set at $p<0.05$.

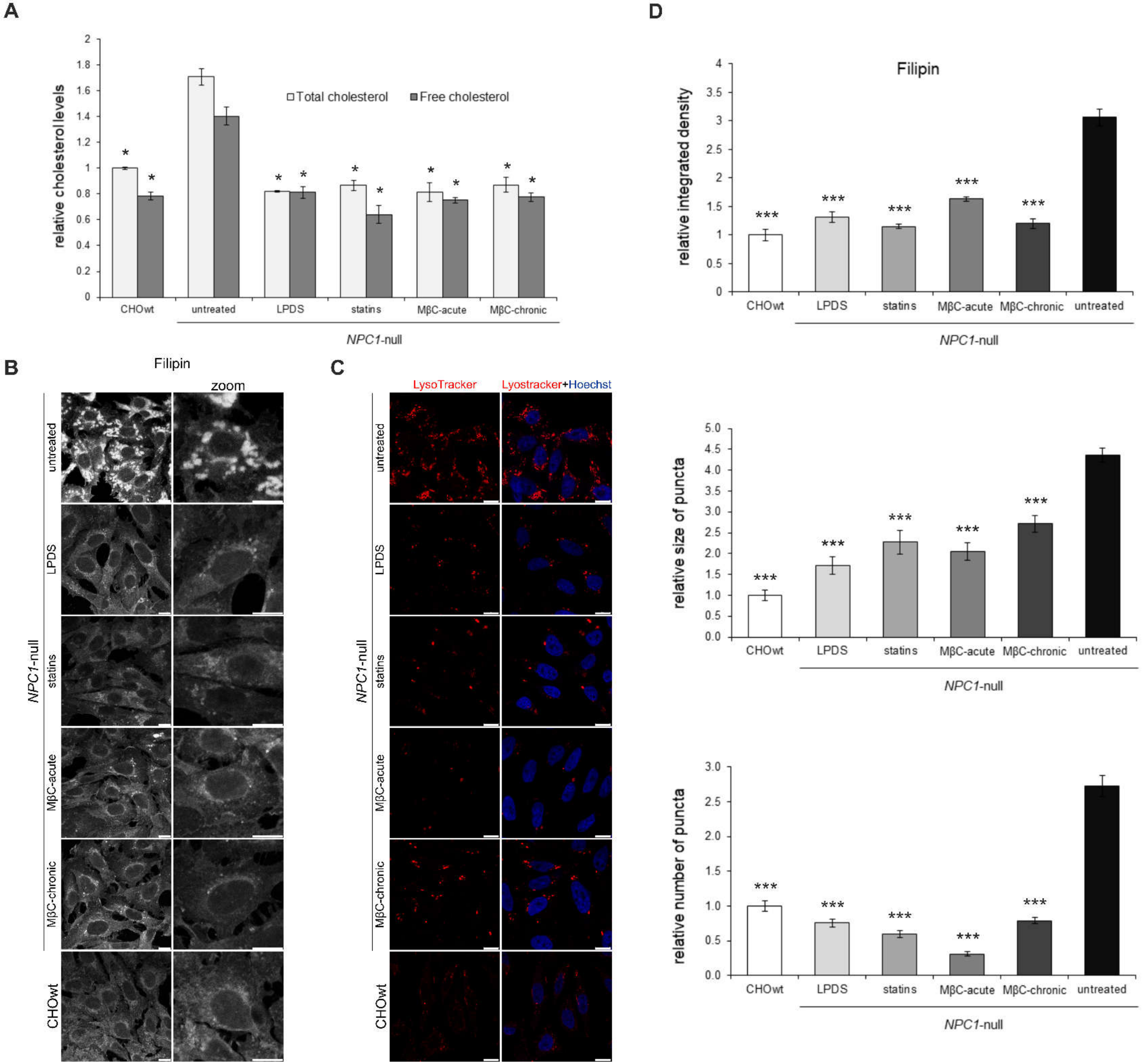
A
relative cholesterol levels
Total cholesterol
Free cholesterol
CHOwt
untreated
LPDS
statins
MβC-acute
MβC-chronic
NPC1-null
B
Filipin
zoom
untreated
LPDS
statins
MβC-acute
MβC-chronic
NPC1-null
CHOwt
C
LysoTracker
Lyostracker+Hoechst
untreated
LPDS
statins
MβC-acute
MβC-chronic
NPC1-null
CHOwt
D
Filipin
relative integrated density
CHOwt
LPDS
statins
MβC-acute
MβC-chronic
untreated
NPC1-null
relative size of puncta
CHOwt
LPDS
statins
MβC-acute
MβC-chronic
untreated
NPC1-null
relative number of puncta
CHOwt
LPDS
statins
MβC-acute
MβC-chronic
untreated
NPC1-null

Figure S4. Cholesterol depletion in *NPC1*-null cells reduces total and free cholesterol levels and rescues lysosomal impairment similar to that as in CHOwt cells. To deplete cholesterol in *NPC1*-null cells, the following treatmens were used: 10% lipoprotein deficient serum (LPDS), statin treatment (4 µM lovastatin and 0.25 mM mevalonate) and methyl-β-cyclodextrin (MβC) to acutely or chronically deplete cholesterol. A**)** The levels of total and free cholesterol in the cell lysates were determined using the Amplex Red Cholesterol Assay kit and were compared to that in *NPC1*-null untreated cells, ***p<0.001. B**)** Filipin was used to stain free cholesterol. C**)** To analyze lysosomal function, live cells grown on glass coverslips were incubated with LysoTracker Red DND-99. Cells were analyzed by confocal microscopy and filipin and Lysotracker signals were quantified using ImageJ software. D) The obtained integrated signal intensity of filipin staining was compared to that in *NPC1*-null untreated cells, ***p<0.001. The size and number of Lysotracker-positive vesicles were quantified and compared to *NPC1*-null untreated cells, ***p<0.001. The data are shown as mean ± SD normalized to control, n = 20 cells per group from three independent experiments. Scale bar – 20 µm (unzoomed figures) and 10 µm (zoomed figures).

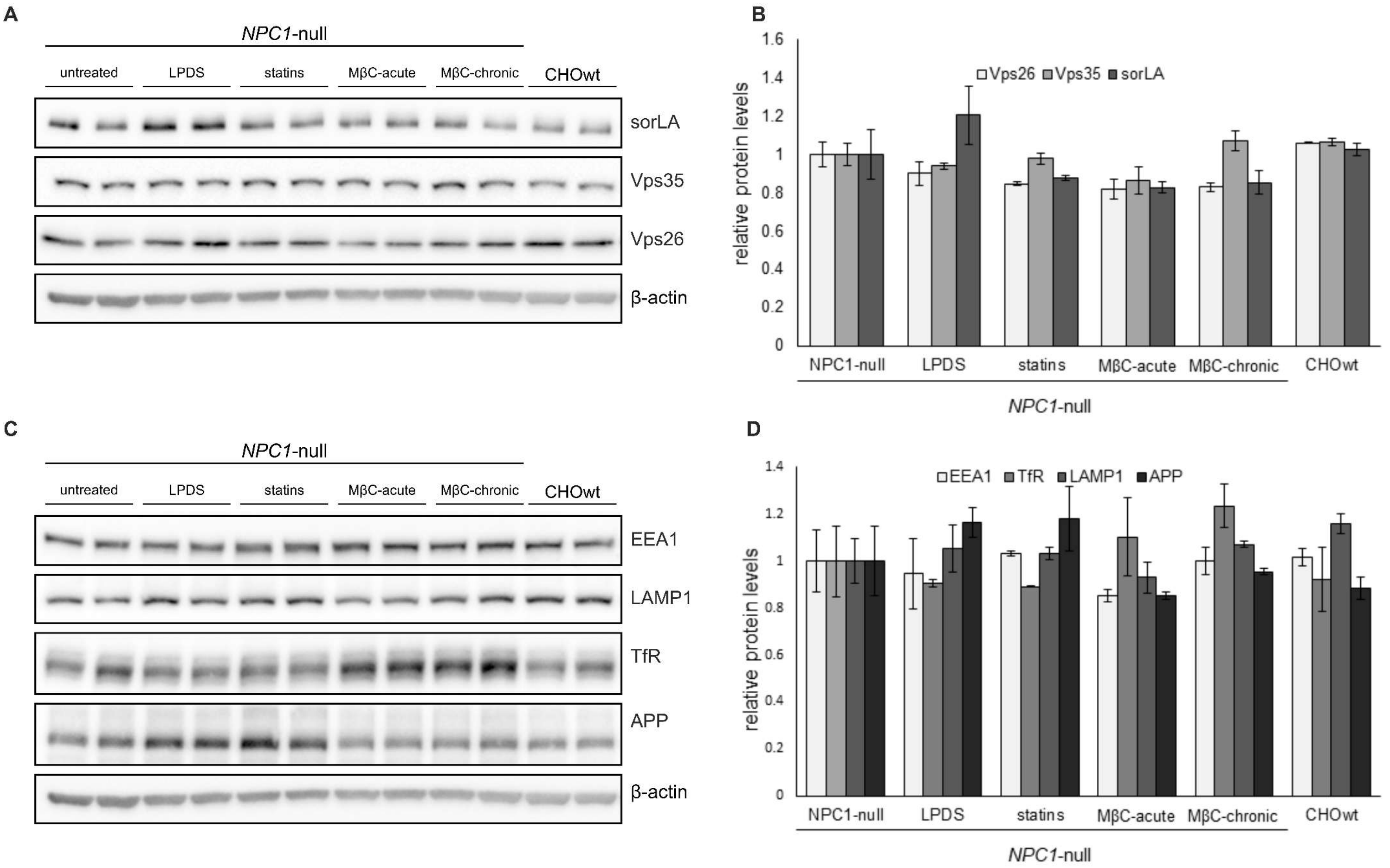
A
NPC1-null
untreated
LPDS
statins
MβC-acute
MβC-chronic
CHOwt
sorLA
Vps35
Vps26
β-actin
B
Vps26
Vps35
sorLA
relative protein levels
1.6
1.4
1.2
1
0.8
0.6
0.4
0.2
0
NPC1-null
LPDS
statins
MβC-acute
MβC-chronic
CHOwt
NPC1-null
C
NPC1-null
untreated
LPDS
statins
MβC-acute
MβC-chronic
CHOwt
EEA1
LAMP1
TfR
APP
β-actin
D
EEA1
TfR
LAMP1
APP
relative protein levels
1.4
1.2
1
0.8
0.6
0.4
0.2
0
NPC1-null
LPDS
statins
MβC-acute
MβC-chronic
CHOwt
NPC1-null

Figure S5. The levels of retromer proteins and endolysosomal markers are not changed upon cholesterol depletion in *NPC1*-null cells. To deplete cholesterol in *NPC1*-null cells, the following treatmens were used: 10% lipoprotein deficient serum (LPDS), statin treatment (4 µM lovastatin and 0.25 mM mevalonate) and methyl-β-cyclodextrin (MβC) to acutely or chronically deplete cholesterol. The protein levels of A**)** retromer proteins Vps26, Vps35 and sorLA, and C**)** endolysosomal protein markers – EEA1 for early endosome, transferrin receptor - TfR for recycling endosomes and LAMP1 for lysosome, as well as APP, were not altered by cholesterol depletion in *NPC1*-null cells. B**)** and D**)** Protein signals were quantified using the ImageJ software. The data are shown as mean ± SD normalized to control, from three independent experiments in technical duplicates. The statistical significance of the tests was set at $p<0.05$.

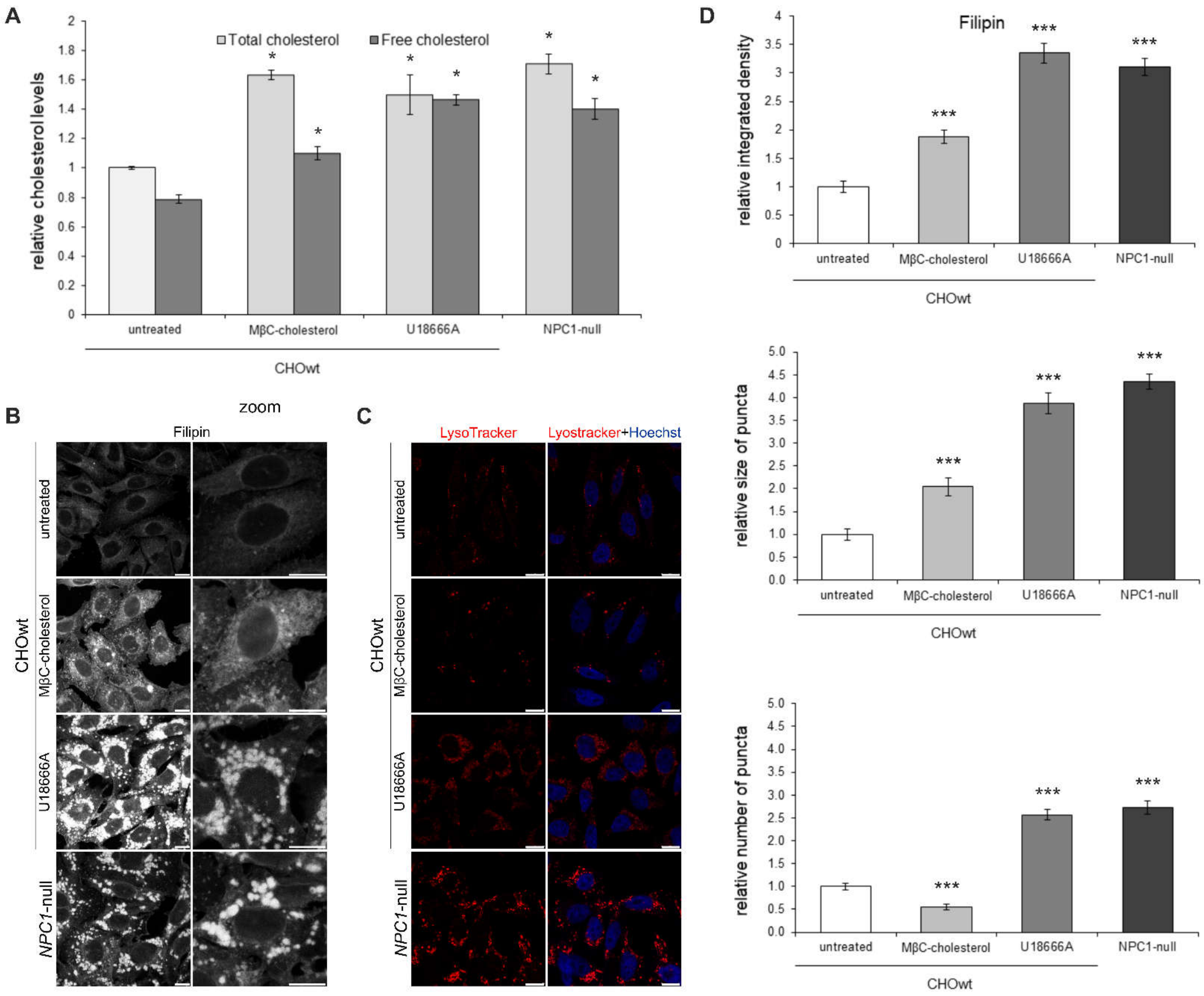
A
relative cholesterol levels
Total cholesterol
Free cholesterol
untreated
MβC-cholesterol
U18666A
NPC1-null
CHOwt
B
zoom
Filipin
untreated
MβC-cholesterol
U18666A
NPC1-null
CHOwt
C
LysoTracker
Lyostracker+Hoechst
D
Filipin
relative integrated density
relative size of puncta
relative number of puncta

Figure S6. Cholesterol loading increased total and free cholesterol levels and caused lysosomal impairment similar to that as in *NPC1*-null cells. To load cholesterol, CHOwt cells were treated with MβC-cholesterol complex and U18666A-compound that mimics NPC phenotype. A**)** The levels of total and free cholesterol in cell lysates were determined using the Amplex Red Cholesterol Assay kit and compared to untreated CHOwt cells, ***$p<0.001$. B**)** Filipin was used to stain free cholesterol. C**)** To analyze lysosomal function, live cells grown on glass coverslips were incubated with LysoTracker Red DND-99. Cells were analyzed by confocal microscopy and filipin and Lysotracker signals were quantified using ImageJ software. D) The obtained integrated signal intensity of filipin staining was compared to that in untreated CHOwt cells, ***$p<0.001$. The size and number of Lysotracker-positive vesicles was quantified and compared to untreated CHOwt cells, ***$p<0.001$. The data are shown as mean ± SD normalized to control, n = 20 cells per group from three independent experiments. Scale bar – 20 µm (unzoomed figures) and 10 µm (zoomed figures).

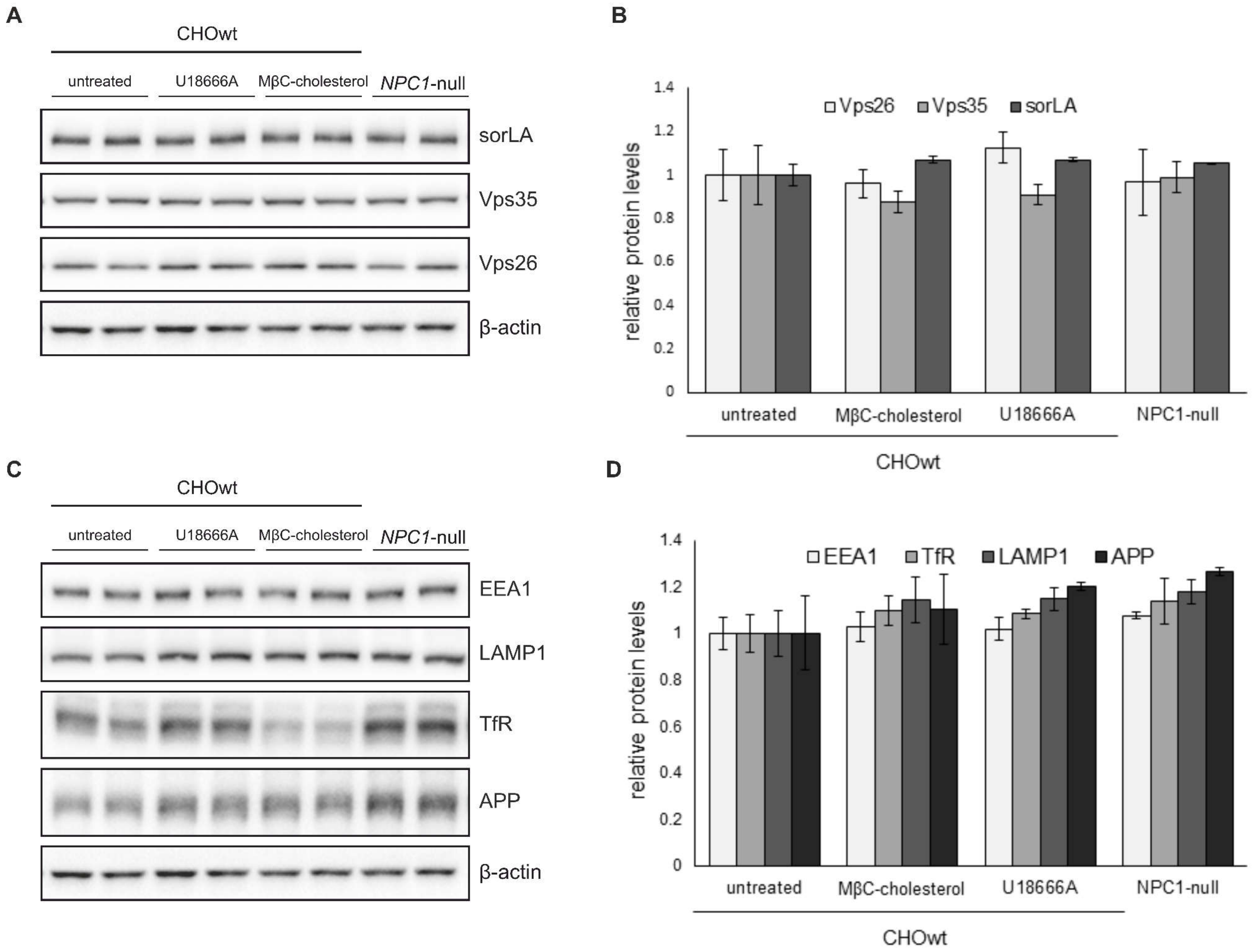

A
CHOwt
untreated
U18666A
MβC-cholesterol
NPC1-null
sorLA
Vps35
Vps26
β-actin
B
Vps26
Vps35
sorLA
relative protein levels
1.4
1.2
1
0.8
0.6
0.4
0.2
0
untreated
MβC-cholesterol
U18666A
NPC1-null
CHOwt
C
CHOwt
untreated
U18666A
MβC-cholesterol
NPC1-null
EEA1
LAMP1
TfR
APP
β-actin
D
EEA1
TfR
LAMP1
APP
relative protein levels
1.4
1.2
1
0.8
0.6
0.4
0.2
0
untreated
MβC-cholesterol
U18666A
NPC1-null
CHOwt

Figure S7. The levels of retromer proteins and endolysosomal markers are not changed upon cholesterol loading in CHOwt cells. To load cholesterol, CHOwt cells were treated with MβC-cholesterol complex and U18666A-compound that mimics NPC phenotype. The protein levels of A**)** retromer proteins Vps26, Vps35 and sorLA, and C**)** endolysosomal protein markers – EEA1 for early endosome, transferrin receptor - TfR for recycling endosomes and LAMP1 for lysosomes, as well as APP, were not altered by cholesterol loading in CHOwt cells. B**)** and D**)** Protein signals were quantified using the ImageJ software. The data are shown as mean ± SD normalized to control, from three independent experiments in technical duplicates. The statistical significance of the tests was set at $p<0.05$.
